# Compact Dual-Quaternion-Based Visual Servoing for Perpendicular Alignment with Surface Normal Constraints

**DOI:** 10.3390/s26061889

**Published:** 2026-03-17

**Authors:** Sheng Li, Chao Ye, Chenlu Liu, Weiyang Lin

**Affiliations:** Research Institute of Intelligent Control and Systems, Harbin Institute of Technology, Harbin 150080, China; lseinstein@163.com (S.L.);

**Keywords:** dual quaternion, visual servoing, manipulator

## Abstract

The ability to reliably press physical buttons is a common requirement in robotics. Conventional vision-based methods often suffer from positional errors during execution if the end-effector’s approach is not perpendicular to the target surface. This paper proposes a novel dual-quaternion-based visual servoing method that enables robots to reach desired poses and enhances accuracy in robotic button-pressing. Our method acquires target pose information (position, depth and surface normal direction) from the RGB-D camera and converts it into dual quaternion representation to construct the visual servoing control system. The image Jacobian matrix for the dual quaternion pose is then computed. The dual-quaternion-based visual servoing ensures that the pressing direction and the optical axis of the coaxially mounted camera remain perpendicular throughout the pressing motion, thereby eliminating misalignment between the actual contact point and the visually identified target. By representing spatial displacements in SE(3) with dual quaternions, our method enables more compact, concise, and efficient pose representation and computation throughout the visual servoing process. Experimental results demonstrate that, compared to conventional methods, our technique achieves more efficient visual servoing control, significantly improving both positioning accuracy and computational efficiency.

## 1. Introduction

The need to reliably locate and press physical buttons represents a pervasive need in numerous robotic application domains. A widely adopted and effective solution in practice employs robots fitted with visual sensors, utilizing advanced computer vision and image recognition algorithms to successfully complete this operation. During the translational movement of the end-effector toward the specific button, if the orientation of the tool is not perpendicular to the plane where the button is located, a positional error will arise between the actual actuation point and the target position identified by image recognition—that is, pressing at an oblique angle will result in a lateral deviation. Therefore, it is necessary to adjust the robot to a pose where the end-effector is positioned straightly above the button and its orientation is orthogonal to the button plane before the execution of the pressing operation.

The application of computer vision provides a viable approach for the autonomous detection, precise localization, and accurate identification of physical buttons, enabling vision-guided robotic interaction and control. Early research primarily relied on traditional image processing techniques. Methods such as edge detection (e.g., Canny operator) [1], shape analysis (e.g., Hough transform), and template matching were widely used for button detection. While effective in structured environments, these approaches were often sensitive to lighting variations, occlusions, and complex backgrounds. Subsequent work integrated feature engineering methods (e.g., SIFT, HOG features) [2,3] with machine learning classifiers (e.g., Support Vector Machines, Random Forests), which improved generalization but still depended on hand-crafted features. In recent years, deep learning-based detection and recognition methods have become the mainstream. Convolutional Neural Networks (CNNs) can automatically learn more discriminative feature representations, significantly enhancing recognition accuracy in complex scenes. Region proposal-based object detection architectures (e.g., Faster R-CNN, YOLO series) [4,5] and instance segmentation models (e.g., Mask R-CNN) [6] have enabled simultaneous button localization, classification, and pixel-level segmentation. Al-Shanoon and Lang [7] demonstrated that CNN-based 3D visual servoing can achieve robust manipulation without requiring precise camera calibration. Ribeiro et al. [8] proposed an end-to-end deep learning approach that directly maps images to robot velocities, achieving real-time performance at 30 fps. Moreover, the introduction of attention mechanisms and Transformer architectures has further improved the model’s ability to focus on critical regions and model long-range dependencies. Wu et al. [9] proposed a hierarchical data-driven predictive control framework for image-based visual servoing systems with unknown dynamics, demonstrating that online learning combined with predictive control can significantly improve tracking accuracy and robustness compared to conventional methods.

Dual quaternions constitute a more advantageous representation for rigid-body motion across multiple dimensions [10]. As a concise and computationally efficient mathematical formalism, dual quaternions enable a unified characterization of spatial transformations—including line geometry mappings—with lower memory requirements and reduced operational costs [11,12,13]. In contrast to traditional methodologies that employ independent control loops for translational and rotational components, dual-quaternion-based techniques naturally incorporate the interdependence between these motions, leading to more physically consistent and intuitive trajectory generation [14]. Furthermore, the combination of screw theory with dual quaternions inherently ensures frame invariance, yielding considerable savings in computational overhead while demanding fewer arithmetic operations for standard tasks such as kinematic composition and exponential calculations. This enhances the performance of both forward and inverse kinematic computations [15]. These beneficial attributes have stimulated growing interest in adopting unit dual quaternions within robot motion planning and control. For example, Wang et al. [16] utilized unit dual quaternion kinematics to formulate a distributed control architecture for networked multi-body systems. Similarly, Daniilidis [17] exploited a dual-quaternion parameterization to concurrently resolve hand-eye rotation and translation via singular value decomposition. Abaunza et al. [18] conducted a systematic comparison of kinematic screw and dual-quaternion-based motion controllers, demonstrating the superior numerical stability of dual quaternion formulations.

Our contribution is to introduce a dual-quaternion-based visual servoing control system designed specifically for tasks requiring precise perpendicular alignment with a surface, such as button pressing. The control system uniquely obtains the target’s pose—including its position, depth, and surface normal direction—from an RGB-D camera and integrates this information into a compact dual quaternion representation. And the corresponding image Jacobian matrix for the dual quaternion pose representation is computed. The dual-quaternion-based visual servoing ensures that the pressing direction coaxial with the optical axis of the end-effector-mounted camera remains perpendicular to the target plane throughout the entire motion, which effectively reduces misalignment between the actual contact point and the visually identified target. By leveraging the mathematical properties of dual quaternions for representing poses in SE(3), our method offers a more compact, concise, and computationally efficient way to construct the visual servoing control system compared to traditional approaches. Unlike image-based pointing systems that explicitly address sensing delays through inertial augmentation [19] or multi-layer controllers that compensate for both sensing dynamics and robot dynamics [20], our method leverages the compact algebraic structure of dual quaternions to achieve computational efficiency with a single-layer geometric control law. While the proposed method does not explicitly incorporate delay compensation or actuator saturation handling, its geometric consistency and reduced computational complexity provide measurable efficiency gains suitable for implementation on various platforms.

This paper is organized as follows: Section 2 provides an overview of the fundamental mathematical framework and operational rules related to dual quaternions. Section 3 proposes the novel method based on unit dual quaternions for robot trajectory planning that yields enhanced accuracy in button-pressing tasks. Section 4 presents the simulation and experimental results to demonstrate the effectiveness and superiority of the proposed method. Section 5 summarized the conclusion.

## 2. Mathematical Background

### 2.1. Quaternion

Several equivalent mathematical forms can represent quaternions. A fundamental form is given by the following definition:

(1) $$q=u+v_{1}i+v_{2}j+v_{3}k,$$

where $u,v_{1},v_{2},v_{3}\in R$ and the imaginary units i,j,k are governed by the following rules:

(2) $$\begin{array}{c} i^{2}=j^{2}=k^{2}=-1, \end{array}$$

(3) $$\begin{array}{c} ij=k,jk=i,ki=j. \end{array}$$

Quaternions extend the concept of complex numbers from a two-dimensional plane to a four-dimensional space. It is also defined as a pair

(4) q=[u,v]

where u∈R is the scalar component, $v\in R^{3}$ is the vector component and $v={\left[v_{1},v_{2},v_{3}\right]}^{⊤}$.

The scalar multiplication of quaternions is

(5) λ[u,v]=[λu,λv]

where λ is an arbitrary scalar.

The conjugate of the quaternion q can be defined as

(6) $$q^{*}=\left[u,-v\right].$$

Let two quaternions be defined as $q_{1}=\left[u_{1},v_{1}\right],q_{2}=\left[u_{2},v_{2}\right]$. The addition and multiplication operations are then given by

(7) $$\begin{array}{c} q_{1}+q_{2}=\left[u_{1}+u_{2},v_{1}+v_{2}\right], \end{array}$$

(8) $$\begin{array}{c} q_{1}q_{2}=\left[u_{1}u_{2}-v_{1}^{⊤}v_{2},u_{1}v_{2}+u_{2}v_{1}+v_{1}\times v_{2}\right]. \end{array}$$

Quaternion multiplication can be represented in matrix form as

(9) $$\begin{array}{ccc} mn & = & \left[\begin{array}{cccc} m_{0} & -m_{1} & -m_{2} & -m_{3}\\ m_{1} & m_{0} & -m_{3} & m_{2}\\ m_{2} & m_{3} & m_{0} & -m_{1}\\ m_{3} & -m_{2} & m_{1} & m_{0} \end{array}\right]\left[\begin{array}{c} n_{0}\\ n_{1}\\ n_{2}\\ n_{3} \end{array}\right]\\ \; & = & H_{m}n\\ \; & = & \left[\begin{array}{cccc} n_{0} & -n_{1} & -n_{2} & -n_{3}\\ n_{1} & n_{0} & n_{3} & -n_{2}\\ n_{2} & n_{3} & n_{0} & -n_{1}\\ n_{3} & n_{2} & -n_{1} & n_{0} \end{array}\right]\left[\begin{array}{c} m_{0}\\ m_{1}\\ m_{2}\\ m_{3} \end{array}\right].\\ \; & = & {\bar{H}}_{n}m \end{array}$$

The norm of a quaternion q=[u,v] is defined as

(10) $$\begin{array}{c} ∥q∥=\sqrt{u^{2}+v_{1}^{2}+v_{2}^{2}+v_{3}^{2}} \end{array}$$

(11) $$\begin{array}{c} =\sqrt{qq^{*}}. \end{array}$$

If ∥q∥≠0, the inverse of a quaternion is given by

(12) $$q^{-1}=\frac{q^{*}}{{∥q∥}^{2}}.$$

Specifically, a quaternion is termed a unit quaternion if its norm is equal to 1. For such a quaternion, its inverse is directly given by the conjugate

(13) $$q^{-1}=q^{*}.$$

Furthermore, a unit quaternion q that represents a rotation by an angle θ about the rotation axis $s\in R^{3}$ can be expressed in the form:

(14) $$q=\left[\cos\frac{\theta }{2},\sin\frac{\theta }{2}s\right]=\cos\frac{\theta }{2}+\sin\frac{\theta }{2}s.$$

### 2.2. Dual Quaternion

The dual number, which is invented by Clifford and developed by Study, is defined as

(15) $$\tilde{w}=m+\varepsilon n\;,\;\mathrm{with}\;\varepsilon^{2}=0,\;\varepsilon \neq 0,$$

where m∈R is called the real part, n∈R is called the dual part and ε is the dual element.

The dual quaternion is constructed by applying the principles of dual numbers to quaternion algebra. Specifically, it can be represented as a quaternion whose components are themselves dual numbers, or equivalently, as a dual number whose two components are quaternions. This leads to the following standard notation:

(16) $$\begin{array}{ccc} \hat{q} & = & q_{r}+\varepsilon q_{d}, \end{array}$$

where $q_{r}$ and $q_{d}$ are quaternions.

The conjugate of a dual quaternion $\hat{q}$ is given by:

(17) $$\begin{array}{ccc} {\hat{q}}^{*} & = & q_{r}^{*}+\varepsilon q_{d}^{*}. \end{array}$$

Let ${\hat{q}}_{1}$ and ${\hat{q}}_{2}$ be two dual quaternions. The addition and multiplication operations are then given by

(18) $$\begin{array}{ccc} {\hat{q}}_{1}+{\hat{q}}_{2} & = & q_{r1}+q_{r2}+\varepsilon \left(q_{d1}+q_{d2}\right), \end{array}$$

(19) $$\begin{array}{ccc} {\hat{q}}_{1}{\hat{q}}_{2} & = & q_{r1}q_{r2}+\varepsilon \left(q_{r1}q_{d2}+q_{d1}q_{r2}\right). \end{array}$$

If $q_{r}\neq 0$, the norm of the dual quaternion $\hat{q}=q_{r}+\varepsilon q_{d}$ is defined as $∥\hat{q}∥=\sqrt{{\hat{q}}^{*}\hat{q}}=\sqrt{\hat{q}{\hat{q}}^{*}}$, and we can obtain

(20) $$\begin{array}{ccc} ∥\hat{q}∥ & = & ∥q_{r}∥+\varepsilon \left(\frac{q_{d}q_{r}^{*}+q_{r}q_{d}^{*}}{2∥q_{r}∥}\right). \end{array}$$

If $∥\hat{q}∥=1$, the dual quaternion $\hat{q}$ is called unit dual quaternion, where the necessary and sufficient conditions are as follows.

A dual quaternion $\hat{q}$ is a unit dual quaternion, defined by $∥\hat{q}∥=1$, if and only if the following conditions hold:

(21) $$\begin{array}{c} q_{d}q_{r}^{*}+q_{r}q_{d}^{*}=0 \end{array}$$

(22) $$\begin{array}{c} ∥q_{r}∥=1. \end{array}$$

And the inverse of a unit dual quaternion is

(23) $${\hat{q}}^{-1}={\hat{q}}^{*}.$$

## 3. Dual Quaternion Visual Servoing

To ensure actuation accuracy when pressing the target button, the tool’s Z-axis must be aligned perpendicular to the button plane before the pressing motion. A misalignment between the tool axis and the surface normal vector will introduce a lateral positional error during the downward motion, causing the actual press point to deviate from the the image recognition results. Therefore, the end-effector must first be moved to a pose where its orientation is normal to the button plane and its position is directly above the target point along the surface normal vector.

### 3.1. Desired Dual Quaternion Pose

Given a plane specified by its unit normal vector n and the Cartesian position coordinate p of a target button, the set of rigid body poses restricted to lie on the normal line through p and to have their orientations aligned with n constitutes a subgroup of SE(3). This subgroup can be parameterized by the unit dual quaternion ${\hat{q}}_{\left(n,p\right)}$.

In terms of the task, a pose satisfies the task requirements if its position is obtained by translating the target position $p_{d}$ along the unit normal vector n by an arbitrary distance *d*, and its orientation coincides with the unit normal vector n while allowing arbitrary rotation about n.

Let $q_{d}$ denote the desired orientation of the end-effector for pressing the button in the world frame. Therefore, the orientation can be described in the form of a unit dual quaternion ${\hat{q}}_{r}$ constructed with $q_{d}$:

(24) $$\begin{array}{ccc} {\hat{q}}_{r} & = & q_{d}+\varepsilon \cdot 0=q_{d} \end{array}$$

Similarly, given a translation vector $m\in R^{3}$ and a pure quaternion t=[0,m], a translation motion can be described as a unit dual quaternion ${\hat{q}}_{t}$ constructed with t:

(25) $$\begin{array}{c} {\hat{q}}_{t}=1+\varepsilon \cdot \left[0,\frac{1}{2}m\right]=1+\frac{1}{2}\varepsilon t. \end{array}$$

If the current Cartesian position coordinate of the end-effector is expressed as $p_{0}$, the closest position in the subgroup ${\hat{q}}_{\left(n,p\right)}$ from the current end-effector position is the projection of $p_{0}$ onto the line through point $p_{d}$ with the unit direction vector n. And this closest position $p_{c}$ can be written as

(26) $$\begin{array}{c} p_{c}=p_{d}+\left(\left(p_{0}-p_{d}\right)\cdot n\right)n \end{array}$$

According to Equation (25), we can consider $p_{c}$ as a translation vector and construct a corresponding pure quaternion $t_{c}=\left[0,p_{c}\right]$. The closest and feasible translation dual quaternion ${\hat{q}}_{t}$ in the world frame can be derived:

(27) $$\begin{array}{ccc} {\hat{q}}_{t} & = & 1+\frac{1}{2}\varepsilon t_{c}. \end{array}$$

Therefore, combining the rotation and translation parts above, a desired pose with closest position and feasible orientation can be described in the form of unit dual quaternions as

(28) $$\begin{array}{ccc} {\hat{q}}_{e} & = & {\hat{q}}_{t}{\hat{q}}_{r} \end{array}$$

(29) $$\begin{array}{ccc} & = & \left(1+\frac{1}{2}\varepsilon t_{c}\right)q_{d} \end{array}$$

(30) $$\begin{array}{ccc} & = & q_{d}+\frac{1}{2}\varepsilon t_{c}q_{d} \end{array}$$

The obtained target pose guarantees that the end-effector is positioned directly above the button and oriented orthogonally to the target plane. Initiating the pressing motion from this pose ensures that the end-effector’s movement direction and the optical axis of the coaxially mounted camera remain orthogonal to the button plane, thereby eliminating any lateral displacement error between the actual and visually identified contact points.

### 3.2. Dual Quaternion Error and Control Law

Our approach is based on eye-in-hand position-based visual servoing. This error is defined in a general form as

(31) $$e\left(t\right)=s^{\prime }-s$$

where *s* is a mapping function of current image features (e.g., point, shape, pose, or frequency-domain features) obtained by the camera, and $s^{\prime }$ represents the mapping function of the desired image features. The system is considered to have reached a stable convergence point when this image error e(t) is driven to zero.

If a camera mounted on the end-effector is used for visual recognition of a fixed target object, the relative pose between the camera and the object can be utilized as the image feature *s*. For notational clarity, we define the current camera frame as frame *c*, the desired camera frame be frame $c^{\prime }$, and the target object frame be frame *s*. The pose transformation of the target object frame with respect to frame *c* and frame $c^{\prime }$, denoted as q^sc and q^sc′ respectively, is represented using dual quaternions.

For a unit quaternion q=[u,v], both q and −q represent the same physical rotation. To ensure uniqueness in the additive error computation, we enforce the convention that the scalar part satisfies u≥0. This is achieved by the mapping: To ensure uniqueness in error computation, we adopt the convention that the scalar part of the quaternion satisfies u≥0. This is implemented as follows: if the scalar part u≥0, the quaternion is used directly; if u<0, we take its negative. This mapping yields a normalized quaternion with a non-negative scalar part.

For a unit dual quaternion $\hat{q}=q_{r}+\varepsilon q_{d}$, the sign ambiguity is resolved by applying the above sign convention to select $q_{r}$. This guarantees a unique representation.

Therefore, we can define the dual quaternion error $\hat{e}$ as the difference between the actual and expected target poses in the camera frame. And the error $\hat{e}$ can be expressed as

(32) e^=q^sc′−q^sc.

The objective of visual servoing control is to move the robot’s end-effector with the camera and minimize the image error $\hat{e}$.

Limited by the camera’s field of view, the visual servoing task operates in a neighborhood of the desired pose where the rotation angle error |θ|<π/2. This ensures that the error remains valid and the system avoids the related singularity. The additive form avoids the quaternion multiplication and logarithm computation required for the relative error, reducing the control loop latency facilitating straightforward proportional control without the need for nonlinear error projection. For the usual task with typical initial poses within 90° of the target and limitation of the camera’s field of view, the additive error provides an feasible approximation.

Let vcc∈R3 and ωcc∈R3 denote the instantaneous linear and angular velocities of the camera expressed in the camera coordinate frame respectively. If the camera’s pose change rate is compactly represented as a vector ξcc=[vc⊤c,ωc⊤c]⊤, the rate of change in the image features q^sc˙, can be expressed as a function of the camera velocity ξcc as follows:

(33) q^sc˙=Lsξcc

where $L_{s}\in R^{8\times 6}$ is the image Jacobian matrix.

Differentiating Equation (32) and substituting Equation (33) into the result, we obtain

(34) e^˙=−Lsξcc.

If we choose the velocity of the camera ξcc as

(35) ξcc=Ls⊤Kce^,

where $K_{c}$ is a positive definite diagonal gain matrix that is manually designed. The choice leads to a linear system.

In the following, we provide a proof of stability for the control system constructed as described above. We define the following Lyapunov function candidate which is a positive definite quadratic function:

(36) $$\begin{array}{c} V\left(\hat{e}\right)=\frac{1}{2}{\hat{e}}^{⊤}K_{c}\hat{e}. \end{array}$$

Differentiating Equation (36) and substituting Equation (34) into the result, we can obtain

(37) V˙(e^)=12e^˙⊤Kce^+12e^⊤Kce^˙=−12ξ˙c⊤cLs⊤Kce^−12e^⊤KcLsξ˙cc.

Since $K_{c}$ is a diagonal matrix and we choose ξcc=Ls⊤Kce^, substituting Equation (35) into Equation (37) derives

(38) $$\begin{array}{c} \dot{V}\left(\hat{e}\right)=-{\hat{e}}^{⊤}K_{c}L_{s}L_{s}^{⊤}K_{c}\hat{e}. \end{array}$$

Therefore, if $K_{c}$ is a positive definite matrix, the derivative of the Lyapunov function of the system is negative. The system is asymptotically stable.

### 3.3. Image Jacobian Matrix Derivation

We now calculate the image Jacobian matrix $L_{s}$. Let the position of the image target expressed in the camera coordinate frame be denoted as a position vector psc∈R3 and a position quaternion tsc=[0,psc] and the orientation be denoted as a quaternion qsc. The pose of the image target described as a unit dual quaternion q^sc can be expressed as

(39) q^sc=u^tu^r=qsc+12εtscqsc=qr+εqd.

We begin by considering the real part of the dual quaternion. Its derivative is computed as follows. The orientation at time *t* and t+Δt is given by the quaternions qsc(t) and qsc(t+Δt), respectively. If we denote the instantaneous angular velocity of the image target expressed in the camera frame as ωsc=[ωx,ωy,ωz]⊤, the rotational change Δq during Δt corresponds to a rotation by an angle θ=∥ωsc∥Δt about the instantaneous axis n=ωsc∥ωsc∥, and is described by

(40) Δq=cosθ2+nsinθ2=cos∥ωsc∥Δt2+ωsc∥ωsc∥sin∥ωsc∥Δt2

It follows that qsc(t+Δt)=qsc(t)Δq. If we consider qsc=[qω,qx,qy,qz]⊤=qsc(t) as the original orientation, we can calculate the quaternion derivative through the difference between qsc(t) and qsc(t+Δt) as

(41) qsc(t+Δt)−qsc(t)=qsccos∥ωsc∥Δt2+ωsc∥ωsc∥sin∥ωsc∥Δt2−qsc=qsc−2sin2∥ωsc∥Δt4+ωsc∥ωsc∥sin∥ωsc∥Δt2.

Therefore, the time-derivative of the quaternions can be obtained

(42) q˙sc=limΔt→0qsc(t+Δt)−qsc(t)Δt=limΔt→01Δtqsc−2sin2∥ωsc∥Δt4+ωsc∥ωsc∥sin∥ωsc∥Δt2=qscωsc∥ωsc∥limΔt→01Δtsin∥ωsc∥Δt2=qscωsc∥ωsc∥ddtsin∥ωsc∥2tt=0=12qscωsc=12−ωxqx−ωyqy−ωzqzωxqw+ωzqy−ωyqzωyqw−ωzqx+ωxqzωzqw+ωyqx−ωxqy.

Finally, we consider the derivative of the pose represented by the unit dual quaternion. Differentiating Equation (39) with respect to time *t*, we can obtain

(43) q^sc˙=12qscωsc+ε12vscqsc+14tscqscωsc.

By substituting the quaternion product given in Equation (9) into Equation (43) and simplifying the result to the matrix form, we can obtain

(44) q^sc˙=012Hqr12H¯qr12Hqdvscωsc=Jdqvscωsc.

Based on the geometric relationship, the velocity of the target object in the camera frame is related to the camera’s velocity as follows:

(45) vsc=−vcc−ωcc×psc

(46) ωsc=−ωcc,

where vcc and ωcc are the linear velocity and angular velocity of the camera in the camera frame respectively.

Similarly, simplifying the result to the matrix form, we can derive

(47) vscωsc=01×301×3−I3[psc]×01×301×303−I3vccωcc=Jcvccωcc,

where [psc]× is the skew-symmetric matrix of the vector psc.

By combining Equation (44) with Equation (47), the image Jacobian matrix $L_{s}$ in Equation (33) can be derived

(48) $$\begin{array}{c} L_{s}=J_{dq}J_{c}. \end{array}$$

**Figure 1:**
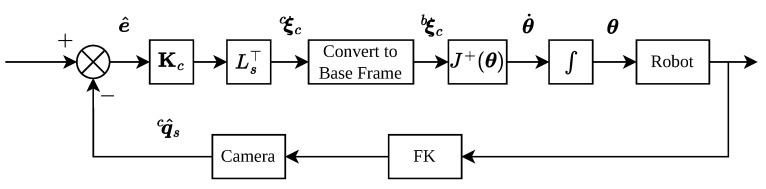
Dual quaternion visual servoing control system.

The control system structure is shown in Figure 1 and the whole control process is shown in Algorithm 1.
**Algorithm 1** Dual-Quaternion-Based Visual Servoing with Normal Alignment**Input:** Gain matrix $K_{c}\in R^{8\times 8}$, Convergence threshold $\epsilon_{th}$ **Output:** Robot reaches pose with end-effector perpendicular to target surface
  1:**Initialize:** Robot current pose ${\hat{q}}_{0}$  2:**while**$\;∥\hat{e}∥>\epsilon_{th}$ **do**  3:   **Perception:**  4:   Capture RGB image and depth image  5:   Detect target position $p_{d}$ and estimate surface normal n  6:   **Desired Pose Construction:**  7:   Compute closest point on normal line: $p_{c}\leftarrow p_{d}+\left(\left(p_{0}-p_{d}\right)\cdot n\right)n$  8:   Construct translation dual quaternion: ${\hat{q}}_{t}\leftarrow 1+\varepsilon \cdot \frac{1}{2}t_{c}$ where $t_{c}=\left[0,p_{c}\right]$  9:   Construct rotation dual quaternion: ${\hat{q}}_{r}\leftarrow q_{d}+\varepsilon \cdot 0$10:   Desired pose: ${\hat{q}}_{e}\leftarrow {\hat{q}}_{t}{\hat{q}}_{r}=q_{d}+\varepsilon \cdot \frac{1}{2}t_{c}q_{d}$11:   **Error Computation:**12:   Ensure scalar part of ${\hat{q}}_{r}$ is non-negative13:   Compute dual quaternion error: e^←q^sc−q^sc′14:   **Control Law:**15:   Compute image Jacobian: $L_{s}\leftarrow J_{dq}J_{c}$16:   Camera velocity: ξcc←Ls⊤Kce^17:   **Execution:**18:   Transform to robot joint velocities: θ˙←J+(θ)·ξcc19:   Send to robot controller and update pose20:**end while**21:**return** Robot reaches pose

## 4. Simulations and Experiments

**Figure 2:**
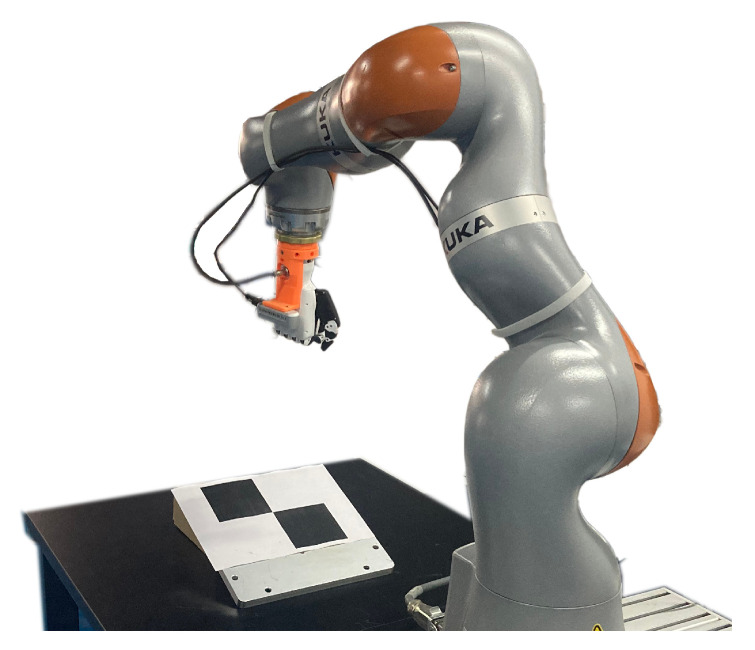
The KUKA LBR iiwa 14 manipulator with Realsense D435i camera.

The experiments were conducted using a KUKA LBR iiwa 14 R820 manipulator (KUKA Deutschland GmbH, Augsburg, Germany). A RealSense Depth D435i camera (Intel Corporation, Santa Clara, CA, USA) which is an RGB-D camera is chosen to be the simulation camera for 3D scene reconstruction, depth information extraction and the image recognition. The RGB-D camera is set on the end-effector, aligned parallel to it. The experimental platform is shown in Figure 2. It is used for image recognition of the target and responsible for acquiring depth information around the target and measuring the normal vector of the plane on which the target is located. The camera intrinsics are set as follows: the image resolution is 1920 × 1080 pixels, the principal point is located at (966, 532), and the focal lengths in the x and y directions are both 1395 pixels. By integrating the depth information and button position data obtained from the camera, the three-dimensional position vector of the target button in the camera coordinate system, along with the normal vector of its residing plane, can be accurately computed. The initial pose of the camera on the robot in the base frame is represented using dual quaternions as

(49) q^sb=[0,0,1.00,0,−0.05,−0.40,0,0.25]⊤.

And the target is selected as a 0.4 m by 0.4 m black and white checkerboard, with its center at ${\left[0.6,0,0.5\right]}^{⊤}$, and its plane normal vector is ${\left[-0.5,0,1\right]}^{⊤}$.

**Figure 3:**
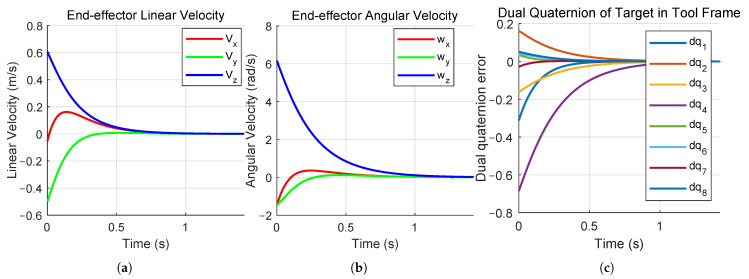
Proposed method simulation results: (**a**) the end-effector linear velocity of the end-effector; (**b**) the end-effector angular velocity of the end-effector; (**c**) the dual quaternion error in the camera frame.

**Figure 4:**
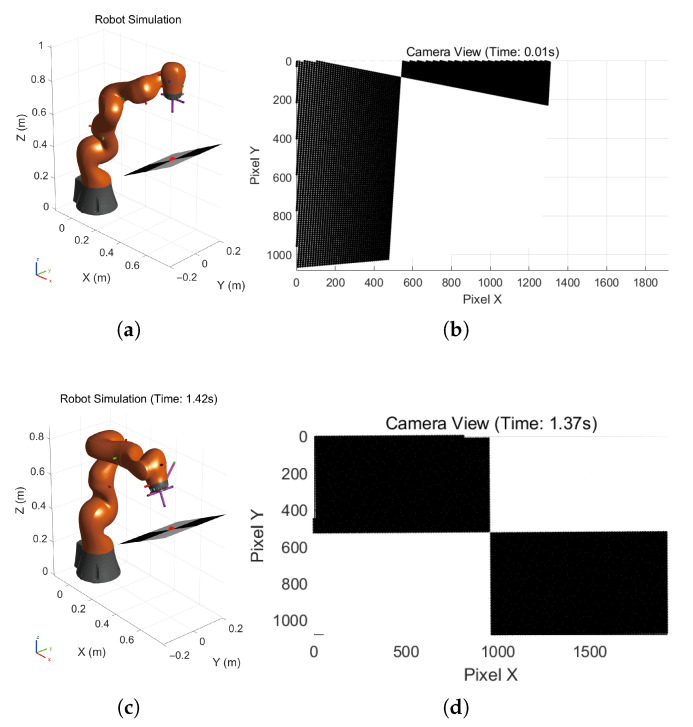
Robot motion and corresponding camera views: (**a**) the initial pose of the robot; (**b**) the initial camera view; (**c**) the final pose of the robot; (**d**) the final camera view.

The simulation results of the dual-quaternion-based visual servoing are presented in Figure 3 and Figure 4. Figure 3a,b illustrate the linear and angular velocities, respectively, of the robot’s end-effector expressed in its own coordinate frame. Both velocities can be seen to converge to zero as the camera and end-effector approach their desired poses. Figure 3c illustrates the pose error between the actual and expected target poses in the camera frame expressed as unit dual quaternions. Figure 4a,b show the initial pose of the robot and the initial camera view, respectively. Figure 4c,d show the final robot pose after task completion and the corresponding camera view. The robot finally moves to the desired pose directly above the target point on the black and white checkerboard, with the end-effector frame oriented perpendicularly to the target plane, indicating that the visual servoing task was accomplished. The pose error converges to ${\left[0,0,0,0,0,0,0,0\right]}^{⊤}$, which means the actual robot pose coincides with the desired pose. By leveraging the mathematical properties of dual quaternions in SE(3), our method offers a compact and computationally efficient visual servoing formulation. Simulation results demonstrate that this approach not only simplifies the system construction but also achieves reliable convergence.

**Figure 5:**
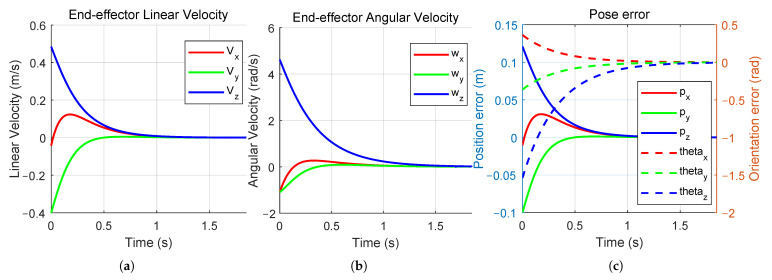
Homogeneous matrix method simulation results: (**a**) the end-effector linear velocity of the end-effector; (**b**) the end-effector angular velocity of the end-effector; (**c**) the pose error in the camera frame.

To quantitatively evaluate the advantages of the proposed dual-quaternion-based approach, we implemented a comparative experiment using the conventional homogeneous transformation matrix method. The same proportional control law with identical gain matrices is applied to ensure a fair comparison. Figure 5 presents the simulation results obtained using the homogeneous matrix method under identical experimental conditions. As shown in Figure 5a,b, the linear and angular velocities of the end-effector exhibit similar convergence trends. Figure 5c reveals the pose error achieved by the homogeneous matrix method are comparable in magnitude but require approximately 1.5 s to converge, whereas the proposed dual quaternion method achieves convergence within approximately 1.2 s.

Table 1 provides a quantitative comparison of computational efficiency between the proposed dual quaternion method and the conventional homogeneous matrix method. The results demonstrate that the proposed method requires only 1.8628 ms per control cycle on average, achieving a 27.9% reduction in computation time compared to the homogeneous matrix method (2.5836 ms). Furthermore, the proposed method exhibits lower computational complexity with 45 FLOPs per control cycle on average versus 65 FLOPs for the homogeneous matrix approach. These gains stem from the compact algebraic structure of dual quaternions, which naturally couples rotational and translational components without requiring separate matrix operations for pose composition and error computation. The reduced computational overhead is particularly advantageous for real-time implementation on resource-constrained robotic platforms.

## 5. Conclusions and Future Work

In this paper, we have introduced a novel visual servoing method based on dual quaternions to fulfill the requirements of reaching poses guaranteeing perpendicular alignment in robotic button-pressing tasks. By formulating the pose representation and error minimization within the dual quaternion algebra, we achieve a compact and computationally efficient approach to conduct the visual servoing control. Our contribution lies in the derivation of the control law and the corresponding image Jacobian matrix for the dual quaternion pose representation, which simplifies the computation and ensures convergence. Although our method effectively decreases execution misalignment in the button-pressing progress, the framework itself is general and applicable to a broad range of precision manipulation tasks. Experimental results show that our dual-quaternion-based approach not only enhances positioning accuracy but also achieves superior computational efficiency compared to conventional methods.

While the proposed method demonstrates effective performance, several practical constraints should be acknowledged. In practice, RGB-D frame acquisition and target detection introduce latency that may affect stability at high speeds. Future work will incorporate delay-compensated control. Furthermore, surface normal estimation from depth data is sensitive to sensor noise, particularly on specular or textureless surfaces. This limits application to surfaces with particular reflecting properties. Robust filtering or learning-based methods could improve this. Moreover, the method assumes the target remains within the camera field of view during servoing. Large initial misalignments may cause target loss, necessitating visual tracking or exploratory motions. For larger workspaces, adjusting camera viewpoint with a proper algorithm would be necessary.

## Figures and Tables

**Table 1:** Computation cost and FLOPS results.

	Proposed Method	Homogeneous Matrix Method
Time per Control Cycle	1.8628 ms	2.5836 ms
FLOPs per Control Cycle	45	65

## Data Availability

The data presented in this study will be available on request from the authors.

## References

[1] Mahalle A.G., Shah A.M. An Efficient Design for Canny Edge Detection Algorithm Using Xilinx System Generator. Proceedings of the 2018 International Conference on Research in Intelligent and Computing in Engineering (RICE).

[2] Zheng L., Yang Y., Tian Q. (2018). SIFT Meets CNN: A Decade Survey of Instance Retrieval. IEEE Trans. Pattern Anal. Mach. Intell..

[3] Surasak T., Takahiro I., Cheng C.H., Wang C.E., Sheng P.Y. Histogram of oriented gradients for human detection in video. Proceedings of the 2018 5th International Conference on Business and Industrial Research (ICBIR).

[4] Yang L., Zhong J., Zhang Y., Bai S., Li G., Yang Y., Zhang J. (2023). An Improving Faster-RCNN with Multi-Attention ResNet for Small Target Detection in Intelligent Autonomous Transport with 6G. IEEE Trans. Intell. Transp. Syst..

[5] Li J., Tang H., Li X., Dou H., Li R. (2024). LEF-YOLO: A Lightweight Method for Intelligent Detection of Four Extreme Wildfires Based on the YOLO Framework. Int. J. Wildland Fire.

[6] Hou M., Huo D., Yang Y., Yang S., Chen H. (2024). Using Mask R-CNN to Rapidly Detect the Gold Foil Shedding of Stone Cultural Heritage in Images. Herit. Sci..

[7] Al-Shanoon A., Lang H. (2022). Robotic Manipulation Based on 3-D Visual Servoing and Deep Neural Networks. Robot. Auton. Syst..

[8] Ribeiro E.G., de Queiroz Mendes R., Grassi V. (2021). Real-Time Deep Learning Approach to Visual Servo Control and Grasp Detection for Autonomous Robotic Manipulation. Robot. Auton. Syst..

[9] Wu J., Jin Z., Liu A., Yu L., Yang F. (2024). A Hierarchical Data-Driven Predictive Control of Image-Based Visual Servoing Systems with Unknown Dynamics. IEEE Trans. Cybern..

[10] Zivkovic N.L., Vidakovic J.Z., Lazarevic M.P. (2023). Forward Kinematics Algorithm in Dual Quaternion Space Based on Denavit-Hartenberg Convention. Appl. Eng. Lett..

[11] Peng X., Shi X., Gong Y. (2019). Dual-Quaternion-Based Modeling and Control for Motion Tracking of a Tumbling Target. J. Syst. Eng. Electron..

[12] Fonseca M.d.P.A., Adorno B.V., Fraisse P. (2020). Coupled Task-Space Admittance Controller Using Dual Quaternion Logarithmic Mapping. IEEE Robot. Autom. Lett..

[13] Thomas F. (2014). Approaching Dual Quaternions From Matrix Algebra. IEEE Trans. Robot..

[14] Han D., Wei Q., Li Z., Sun W. (2008). Control of Oriented Mechanical Systems: A Method Based on Dual Quaternion. IFAC Proc. Vol..

[15] Dantam N.T. (2021). Robust and Efficient Forward, Differential, and Inverse Kinematics Using Dual Quaternions. Int. J. Robot. Res..

[16] Wang X., Han D., Yu C., Zheng Z. (2012). The Geometric Structure of Unit Dual Quaternion with Application in Kinematic Control. J. Math. Anal. Appl..

[17] Daniilidis K. (1999). Hand-Eye Calibration Using Dual Quaternions. Int. J. Robot. Res..

[18] Abaunza H., Chandra R., Özgür E., Corrales Ramón J.A., Mezouar Y. (2022). Kinematic Screws and Dual Quaternion Based Motion Controllers. Control Eng. Pract..

[19] Hurak Z., Rezac M. (2012). Image-Based Pointing and Tracking for Inertially Stabilized Airborne Camera Platform. IEEE Trans. Control Syst. Technol..

[20] Wang C., Lin C.Y., Tomizuka M. (2013). Visual Servoing Considering Sensing Dynamics and Robot Dynamics. IFAC Proc. Vol..

